# Granulocytic myeloid-derived suppressor cells inhibit T follicular helper cells during experimental *Schistosoma japonicum* infection

**DOI:** 10.1186/s13071-021-05006-8

**Published:** 2021-09-26

**Authors:** Yumei Zhang, Yulong Wu, Hua Liu, Wenci Gong, Yuan Hu, Yujuan Shen, Jianping Cao

**Affiliations:** 1 Key Laboratory of Parasite and Vector Biology, National Health Commission of the People’s Republic of China, Shanghai, 200025 China; 2 Department of Pathogenic Biology, Binzhou Medical University, Yantai, Shandong 264003 China; 3 National Institute of Parasitic Diseases, Chinese Center for Disease Control and Prevention (Chinese Center for Tropical Diseases Research), Shanghai, 200025 China; 4 WHO Collaborating Center for Tropical Diseases, Shanghai, 200025 China; 5 National Center for International Research On Tropical Diseases, Ministry of Science and Technology, Shanghai, 200025 China

**Keywords:** *Schistosoma japonicum*, T follicular helper cells, Programmed cell death protein 1, Programmed cell death ligand 1, Myeloid-derived suppressor cells

## Abstract

**Background:**

CD4^+^ T helper (Th) cells play critical roles in both host humoral and cellular immunity against parasitic infection and in the immunopathology of schistosomiasis. T follicular helper (Tfh) cells are a specialized subset of Th cells involved in immunity against infectious diseases. However, the role of Tfh cells in schistosome infection is not fully understood. In this study, the dynamics and roles of Tfh cell regulation were examined. We demonstrated that granulocytic myeloid-derived suppressor cells (G-MDSC) can suppress the proliferation of Tfh cells.

**Methods:**

The levels of Tfh cells and two other Th cells (Th1, Th2) were quantitated at different *Schistosoma japonicum* infection times (0,3, 5, 8, 13 weeks) using flow cytometry. The proliferation of Tfh cells stimulated by soluble egg antigen (SEA) and soluble worm antigen (SWA) in vivo and in vitro were analyzed. Tfh cells were co-cultured with MDSC to detect the proliferation of Tfh cells labelled by 5(6)-carboxyfluorescein diacetate *N*-succinimidyl ester. We dynamically monitored the expression of programmed cell death protein 1 (PD-1) on the surface of Tfh cells and programmed cell death ligand 1 (PD-L1) on the surface of MDSC at different infection times (0, 3, 5, 8 weeks). Naïve CD4^+^ T cells (in Tfh cell differentiation) were co-cultured with G-MDSC or monocytic MDSC in the presence, or in the absence, of PD-L1 blocking antibody.

**Results:**

The proportion of Tfh cells among CD4^+^ T cells increased gradually with time of *S. japonicum* infection, reaching a peak at 8 weeks, after which it decreased gradually. Both SEA and SWA caused an increase in Tfh cells in vitro and in vivo. It was found that MDSC can suppress the proliferation of Tfh cells. The expression of PD-1 on Tfh cells and PD-L1 from MDSC cells increased with prolongation of the infection cycle. G-MDSC might regulate Tfh cells through the PD-1/PD-L1 pathway.

**Conclusions:**

The reported study not only reveals the dynamics of Tfh cell regulation during *S. japonicum* infection*,* but also provides evidence that G-MDSC may regulate Tfh cells by PD-1/PD-L1. This study provides strong evidence for the important role of Tfh cells in the immune response to *S. japonicum* infection.

**Graphical abstract:**

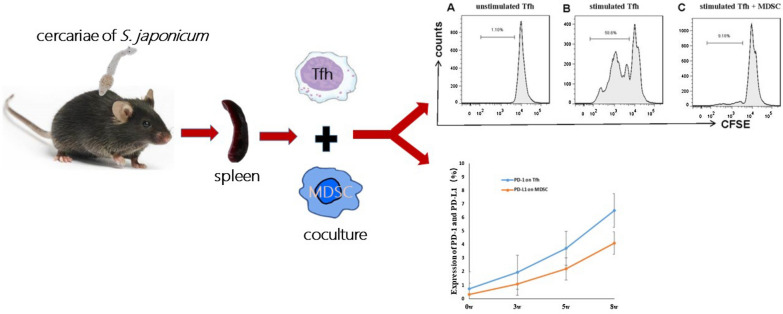

## Background

Schistosomiasis is a neglected public health problem in many developing countries, with more than 200 million people infected worldwide [1–5]. The mechanism of the host immune response to *Schistosoma japonicum* infection is complicated. However, our lack of knowledge of the details of this mechanism represents a research bottleneck for the prevention and control of schistosomiasis and for the development of vaccines against *S. japonicum*. Many immune cells are involved in the immune response to a *S. japonicum* infection, such as T helper type 1 (Th1), Th2, Th17, T follicular helper (Tfh), regulatory T (Treg) cells, dendritic cells (DCs) and myeloid-derived suppressor cells (MDSC).

Tfh cells belong to a subpopulation of CD4^+^ T cells that promote the generation of the germinal center and the production of antibodies by germinal center B cells. Recent evidence shows that Tfh cells may have other functions. For example, the inducible T-cell co-stimulator molecule on the Tfh cell surface can promote liver granuloma formation in mice infected with *S. japonicum* [6]. However, research on Tfh cells and their involvement in the immune response to *S. japonicum* infection is limited and its findings unclear.

A large number of cellular interactions participate in Tfh cell development. For example, DC programmed cell death ligand 1 ((PD-L1) is essential for limiting Tfh cell differentiation [7]. In influenza A virus infections, late activator antigen-presenting cells promote a Tfh response in the lymph nodes that drain the lungs [8]. Additionally, plasma cells are also reported to negatively regulate Tfh cell programming [9]. Moreover, the generation of Tfh cells is driven by macrophages in mice infected with *S. japonicum* [10]. However, little is known about the potential role of MDSC in the inhibition of Tfh cell development in *S. japonicum* infection.

In this study, C57BL/6 mice infected with *S. japonicum* were used as the model to analyse the dynamics and roles of Tfh cells during parasitic infection. Our findings indicate that granulocytic (G)-MDSC might regulate Tfh by programmed cell death protein 1 (PD-1) and PD-L1. The results of this study will contribute to our understanding of the mechanism of the host immune response, and provide a scientific basis for immunization and research on vaccines for schistosomiasis.

## Methods

### Mice and their infection with *S. japonicum*

Female C57BL/6 mice (aged 6–8 weeks) were purchased from SLAC Laboratory (Shanghai, China). All animal experiments were performed in accordance with the Chinese laws for animal protection and in adherence with experimental guidelines and procedures approved by the Institutional Animal Care and Use Committee. *Schistosoma japonicum* (Chinese mainland strain) cercariae were obtained from the National Institute of Parasitic Diseases, Chinese Center for Disease Control and Prevention. For the kinetic analysis of Tfh and MDSC cell populations, each mouse was infected with 20 cercariae of *S. japonicum* through the skin of the abdomen. At 0, 3, 5, 8 and 13 weeks post-infection, five mice were randomly chosen from the infected and normal control groups and sacrificed for further study.

### Antigen preparation and mouse immunization

*Schistosoma japonicum* adult worms were obtained from infected rabbits, and soluble worm antigen (SWA) was prepared from the soluble fraction acquired from adult worms treated by ultrasound, as previously described [11]. The eggs of *S. japonicum* were purified from the livers of infected rabbits and used to prepare *S. japonicum* soluble egg antigen (SEA) [12, 13]. SWA and SEA were diluted to a final concentration of 10 mg/ml in phosphate-buffered saline (PBS). C57BL/6 mice were randomly divided into three groups (control, SEA treated and SWA treated) with five mice in each group. Three independent experiments were carried out following the same methodology. SEA, SWA and PBS were emulsified in incomplete Freund’s adjuvant (IFA; Sigma-Aldrich, ST. Louis, MO) [14]. Each mouse received a subcutaneous injection of 50 μg emulsified SEA, SWA or PBS at the same site in the back; booster injections were given twice at 14-day intervals. Two weeks after the final injection, Tfh levels in spleen cells were measured.

### Cell culture by SEA and SWA stimulation

Splenocytes of C57BL/6 normal mice were cultured in 1 ml complete Roswell Park Memorial Institute (RPMI) 1640 medium containing 10% fetal bovine serum and 100 U of penicillin and streptomycin.

There were three treatment groups: the control group (splenocytes with PBS), the SEA stimulation group (splenocytes with a final concentration of 10 μg/ml SEA) and the SWA stimulation group (splenocytes with a final concentration of 10 μg/ml SWA). Triplicate wells were set up for each group, and 1 × 10^6^ cells per well were cultured in 24-well plates for 72 h at 37 °C. The cells were collected and stained using flow cytometry to detect the proliferation of Tfh cells.

### Flow cytometry analysis

Single-cell suspensions were prepared from the murine spleens at 0, 3, 5, 8 and 13 weeks post-infection. The splenocytes were squeezed by the plunger of a 5-ml syringe through a 70-μm cell strainer. Splenocytes were haemolysed in erythrocyte-lysing buffer and washed in PBS.

To analyse Tfh and MDSC cells, spleen cells were incubated with optimized concentrations of anti-CD4 PE-cy7, anti-mouse CD185 (CXCR5) allophycocyanin (APC), anti-mouse CD279 (PD-1) phycoerythrin (PE), anti-mouse CD11b APC, anti-mouse Gr-1 PerCP-cy5.5, anti-mouse PD-L1-PE. For the detection of Th1 or Th2 cells, single-cell suspensions of splenocytes from each mouse were prepared and stimulated with 50 ng/ml phorbol 12-myristate 13-acetate and 1 μg/ml ionomycin (Sigma-Aldrich) in the presence of 10 μg/ml brefeldin A in complete RPMI 1640 for 6 h at 37 °C. The cells were collected and PE-cy5.5-labelled anti-CD3, and fluorescein isothiocyanate (FITC)-labelled anti-CD4. Subsequently, the cells were permeabilised with BD Cytofix/Cytoperm buffer (BD Biosciences) and PE-labelled anti-IFN-γ and PE-labelled anti-IL-4. All fluorescently labelled antibodies were from eBioscience (San Diego, CA). The data were analyzed using FlowJo7.6 software.

### 5(6)-Carboxyfluorescein diacetate *N*-succinimidyl ester-labelled Tfh cells co-cultured with MDSC

CD4^+^CXCR5^+^ Tfh cells were sorted by flow cytometry from normal C57BL/6 mice splenocytes. CD11b^+^Gr-1^+^ MDSC were sorted from *S. japonicum*-infected C57BL/6 mice splenocytes by the Myeloid-Derived Suppressor Cell Isolation Kit (Miltenyi Biotech, USA).

Freshly prepared Tfh cells were incubated with 5(6)-carboxyfluorescein diacetate *N*-succinimidyl ester (CFSE) (final concentration of 2 μM; Dojindo Laboratory, Japan) for 15 min at 37 °C. Cells were then resuspended and washed in PBS containing 10% fetal bovine serum. CFSE-labelled Tfh cells were divided into three groups: unstimulated, stimulated with anti-CD3 (5 μg/ml; PeproTech, USA) and anti-CD28 (2 μg/ml, PeproTech), stimulated with anti-CD3 and anti-CD28 plus MDSC. Cells were harvested to analyse the CFSE fluorescence intensity of Tfh by flow cytometry after 5 days.

### MDSC co-cultured with naïve CD4^+^ T cells in the Tfh cell differentiation condition

CD4^+^CD44^low^CD62L^high^-naïve T cells were sorted (> 95% pure) from normal C57BL/6 mice splenocytes by EasySep Mouse Naïve CD4^+^ T Cell Isolation Kit (STEMCELL Technologies, Canada). Naïve CD4^+^ T cells were cultured with anti-CD3 (5 μg/ml) and anti-CD28 (2 μg/ml) in the presence of anti-IFN-γ (10 μg/ml; PeproTech), anti-IL-4 (10 μg/ml; PeproTech), and IL-21 (50 ng/ml; PeproTech) in 24-well plates. G-MDSC and monocytic MDSC (M-MDSC) were sorted from *S. japonicum*-infected C57BL/6 mice splenocytes by a Myeloid-Derived Suppressor Cell Isolation Kit (Miltenyi Biotech). G-MDSC or M-MDSC were added to the naïve T cell (5 × 10^5^ cells/well) culture on day 0 at a ratio of 2:1 (MDSC/T cell). Anti-mouse PD-L1 (eBioscience, USA) blocking antibodies and isotypes were used at 5 μg/ml. Cells were cultured in triplicate in RPMI 1640 media for 5 days.

### Statistical analysis

The differences between groups were assessed by one-way ANOVA followed by a least significant difference test using SPSS19.0 software. A *P-*value < 0.05 was considered statistically significant.

## Results

### Dynamics of Th1/Th2/Tfh in mice infected with* S. japonicum*

**Fig. 1a–d:**
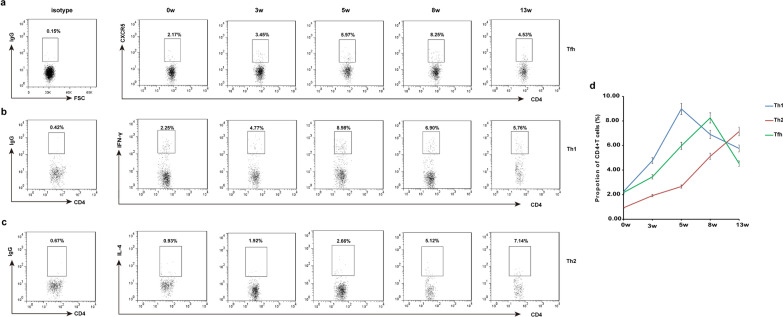
Kinetics of T helper type 1 (*Th1*), Th2 and T follicular helper (*Tfh*) cells in *Schistosoma japonicum* infection. Each mouse was infected with 20 cercariae of *S. japonicum* and five mice were sacrificed at 0 (before infection), 3, 5, 8 and 13 weeks post-infection. Splenocytes were stimulated with phorbol 12-myristate 13-acetate and ionomycin (Sigma-Aldrich) in the presence of brefeldin A in complete Roswell Park Memorial Institute 1640 medium for 6 h. Cells were stained with anti-CD3 PE-cy5.5 and anti-CD4 fluorescein isothiocyanate (FITC) and were then intracellularly stained with anti-IFN-γ PE, anti-IL-4 PE or isotype IgG2a control antibody for the analysis of Th1 or Th2 cells. Single-cell suspensions of splenocytes were stained with anti-CD3 PE-cy5.5, anti-CD4 FITC, anti-CXCR5 allophycocyanin (APC) or isotype IgG2a control antibody for Tfh cells. All of the values were gated on CD3^+^CD4^+^ cells. **a** Kinetics of the percentages of Tfh cells amongst total CD4^+^ T cells from mouse spleens determined using flow cytometry. **b** Kinetics of the percentage of Th1 amongst total CD4^+^ T cells from mouse spleens determined using flow cytometry. **c** Kinetics of percentage of Th2 amongst total CD4^+^ T cells from mouse spleens determined using flow cytometry. **d** Comparison of the dynamics of Th1/Th2/Tfh in mice infected with *S. japonicum*. Data are expressed as the mean ± SD of 25 mice from three independent experiments

Mice were infected with *S. japonicum* and euthanized at 0, 3, 5, 8 and 13 weeks post-infection. Splenocytes were harvested and the levels of Th1/Th2/Tfh specific phenotypic molecules were quantified with flow cytometry (Fig. 1a–c). The proportion of Th1 cells among CD4^+^ T cells increased gradually with the duration of *S. japonicum* infection, reaching a peak at 5 weeks, after which it decreased gradually (Fig. 1d). The proportion of Tfh cells among CD4^+^ T cells increased gradually with duration of *S. japonicum* infection, reaching a peak at 8 weeks, after which it decreased gradually. The proportion of Th2 cells among CD4^+^ T cells increased gradually with duration of *S. japonicum* infection.

### Tfh levels stimulated by SEA and SWA

**Fig. 2a–d:**
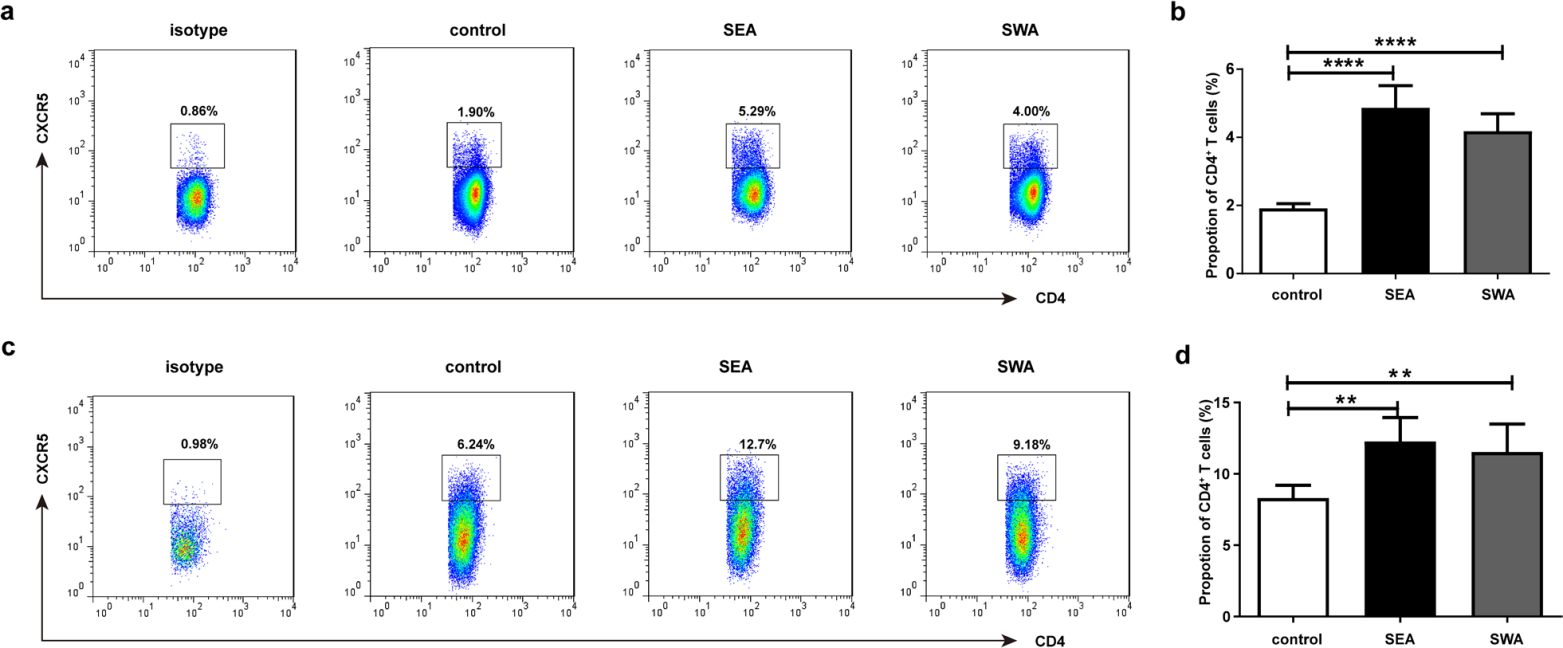
Analysis of Tfh stimulated by soluble egg antigen (*SEA*) and soluble worm antigen (*SWA*). For each of three independent immunization experiments, C57BL/6 mice (five per group) were injected subcutaneously in the back with 200 μl of incomplete Freund’s adjuvant containing 50 μg of SEA, 50 μg of SWA or phosphate-buffered saline (PBS), boosted twice at 14-day intervals. Single-cell splenocyte suspensions of mice were prepared 2 weeks after the final injection. For each of three independent in vitro experiments, splenocytes of C57BL/6 normal mice were stimulated with PBS, final concentration of 10 μg/ml SEA or SWA for 72 h at 37 ℃. The splenocytes were stained with anti-CD4 FITC and anti-CXCR5 APC or isotype IgG2a control antibody for Tfh cells. **a** Analysis of Tfh stimulated by SEA and SWA in vivo by flow cytometry. **b** Comparison of Tfh stimulated by SEA and SWA in vivo*.* Results are expressed as mean ± SD (****P < 0.0001). **d** Analysis of Tfh stimulated by SEA and SWA in vitro by flow cytometry. **d** Comparison of Tfh stimulated by SEA and SWA in vitro (**P < 0.01). Cells were gated on the CD3^+^CD4^+^ population for analysis of Tfh cells.* MDSC* Myeloid-derived suppressor cells,* CFSE* 5(6)-carboxyfluorescein diacetate *N*-succinimidyl ester; for other abbreviations, see Fig. 1

To further study the effects of two *S. japonicum* antigens, SEA and SWA, on Tfh cell generation, C57BL/6 mice were immunized with SWA and SEA. SWA and SEA were also used to induce CD4^+^ T cells to differentiate in vitro. SEA and SWA can cause an increase in Tfh cells both in vivo [*F*_(2, 12)_ = 57.834, *P* < 0.0001; Fig. 2a, b] and in vitro [*F*_(2, 12)_ = 8.921, *P* = 0.004; Fig. 2c, d]. There was no significant difference in the proliferation of Tfh cells between the SEA and SWA treatments.

### MDSC suppressed Tfh cell proliferation

**Fig. 3a–c:**
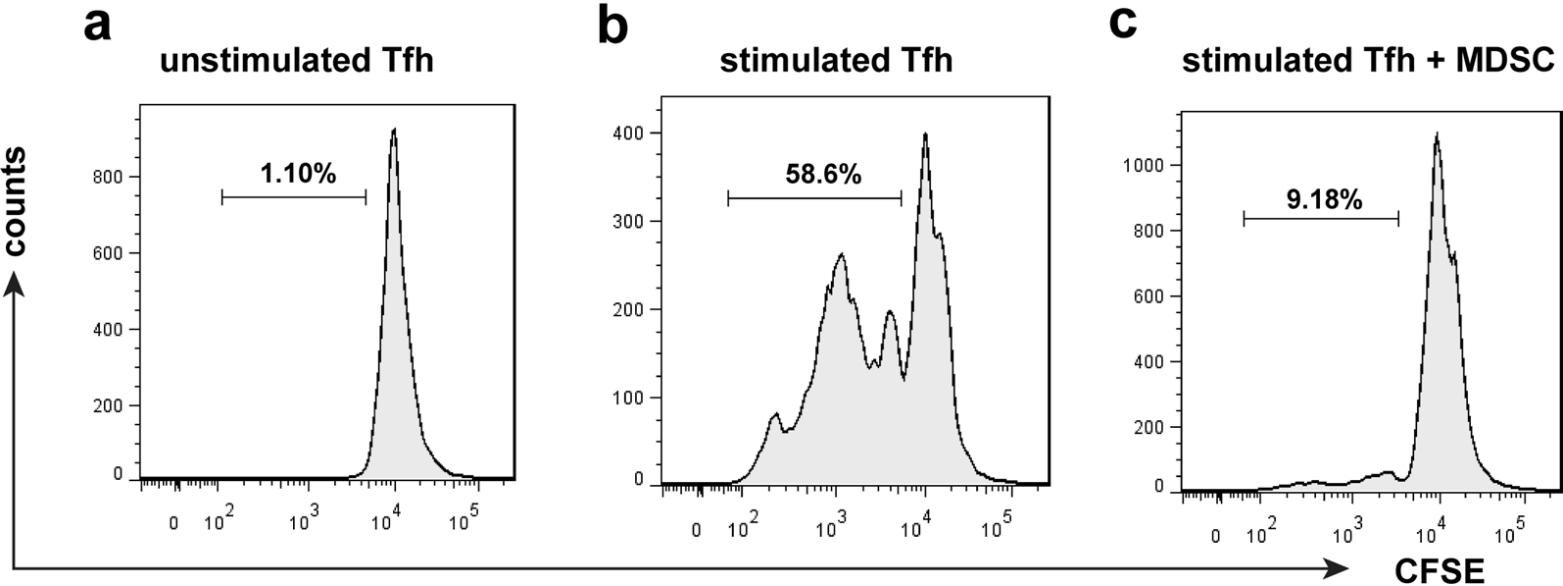
Tfh cell proliferation monitored by CFSE labelling. CD4^+^CXCR5^+^ Tfh cells of C57BL/6 normal mice were sorted by flow cytometry, and CD11b^+^Gr-1^+^ MDSC of *Schistosoma japonicum*-infected C57BL/6 mice were sorted by the Myeloid-Derived Suppressor Cell Isolation Kit. CFSE-labelled Tfh cells (1 × 10^5^/well) were cultured either unstimulated (**a**)**,** stimulated with anti-CD3 (5 μg/ml) and anti-CD28 (2 μg/ml) (**b**)**,** or stimulated with anti-CD3 (5 μg/ml) and anti-CD28 (2 μg/ml) plus MDSC (2 × 10^5^/well) (**c**). Cells were harvested after 5 days to analyse the CFSE fluorescence intensity of Tfh by flow cytometry. For abbreviations, see Figs. 1 and 2

In order to examine Tfh cell proliferative responses to MDSC, we examined CFSE-labelled Tfh cell proliferation by flow cytometry. CFSE-labelled Tfh cells proliferated in response to stimulation (treatment with anti-CD3 antibody combined with anti-CD28 antibody) while unstimulated Tfh cells did not (Fig. 3a, b). The presence of MDSC fully suppressed the proliferation of Tfh cells (Fig. 3c).

### Similar trends observed between the expression of PD-1 on Tfh and PD-L1 on MDSC

**Fig. 4a–c:**
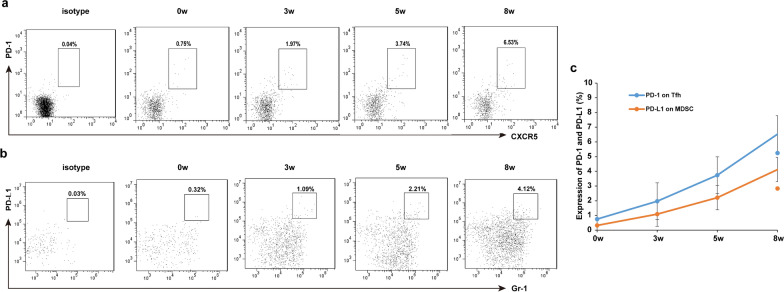
The expression of programmed cell death protein 1 (*PD-1*) on Tfh cells and the expression of programmed cell death ligand 1 (*PD-L1*) on MDSC infected with *Schistosoma japonicum.* Each mouse was infected with 20 cercariae of *S. japonicum* and five mice were sacrificed at 0 (before infection) and 3, 5, and 8 weeks (*wk*) post-infection. Single-cell suspensions of splenocytes were stained with anti-CD4 FITC, anti-CXCR5 APC, anti-PD-1 PE (for Tfh cells) and anti-CD11b APC, anti-Gr-1 PerCP-cy5.5, anti-PD-L1 PE (for MDSC cells). **a** The expression of PD-1 on Tfh cells of mice infected with *S. japonicum*. **b** The expression of PD-L1 on MDSC of mice infected with *S. japonicum.* **c** The expression and trend of PD-1 and PD-L1 at different infection times (0, 3, 5, 8 weeks). Data are expressed as the mean ± SD. For other abbreviations, see Figs. 1 and 2

We interrogated the mechanism of MDSC suppression of Tfh cell proliferation. First, we observed the kinetics of the expression of PD-1 on Tfh cells. Flow cytometric analysis showed that the frequency of PD-1 expression in splenic Tfh cells increased continuously for 8 weeks post-infection compared to that before infection (Fig. 4a). As a receptor of PD-1, the combination of PD-L1 and PD1 can prevent the activation, proliferation and immune effect of T cells. We observed that the frequency of PD-L1 expression in splenic MDSC cells increased continuously for 8 weeks post-infection (Fig. 4b). The expression of PD-1 and PD-L1 increased with the prolongation of the infection cycle. There was a similar trend between the expression of PD-1 on Tfh and the expression of PD-L1 on MDSC in mice infected with *S. japonicum* (Fig. 4c). We speculate that these results provide evidence that MDSC might regulate Tfh via the PD-1/PD-L1 pathway.

### G-MDSC inhibit Tfh cell differentiation through the PD-1/PD-L1 pathway under Tfh cell-polarizing conditions

**Fig. 5:**
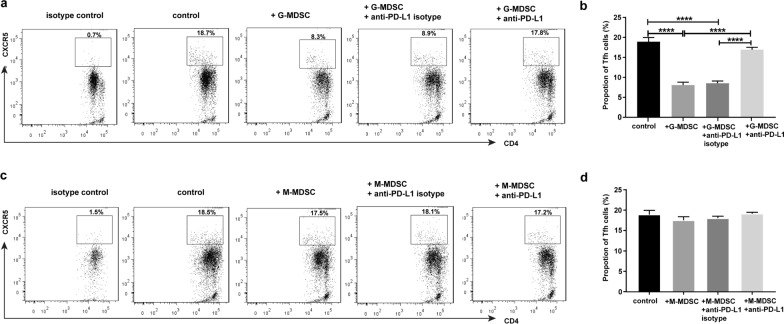
Granulocytic-MDSC (*G-MDSC*) inhibit Tfh cell differentiation through the PD-1/PD-L1 pathway under Tfh-cell-polarizing conditions. CD4^+^CD44^low^CD62L^high^-naïve T cells were sorted from normal C57BL/6 mice splenocytes by the EasySep Mouse Naïve CD4^+^ T Cell Isolation Kit. G-MDSC and M-MDSC were sorted from *Schistosoma japonicum* infected mice by the Myeloid-Derived Suppressor Cell Isolation Kit. Naïve CD4^+^ T cells (5 × 10^5^ cells/well) were co-cultured with G-MDSC or M-MDSC at a ratio of 1:2 (T cell/MDSC) in the presence of anti-CD3, anti-CD28, anti-IFN-γ, anti-IL-4, IL-21 in 24-well plates for 5 days. PD-L1 blocking antibodies and isotypes were used at 5 μg/ml. The percentage of CD4^+^CXCR5^+^ Tfh in different groups (**a** with G-MDSC, **c** with M-MDSC). Comparison of Tfh in different groups (**b** with G-MDSC, **d** with M-MDSC). Data are expressed as the mean ± SD. One-way ANOVA followed by least significant difference test, ****P < 0.0001. For other abbreviations, see Figs. 1, 2 and 4

MDSC in mice are broadly grouped into two subpopulations: G-MDSC and M-MDSC. To determine the inhibitory effect of MDSC on Tfh by PD-1/PD-L1, G-MDSC or M-MDSC were co-cultured with naïve CD4^+^ T cells in vitro for 5 days in the Tfh condition. Strikingly, the presence of G-MDSC greatly reduced the percentage of Tfh cells compared with the control group (without G-MDSC). However, the percentage of Tfh cells was not reduced by G-MDSC in the presence of anti-PD-L1 [*F*_(3, 8)_ = 170.055, *P* < 0.0001; Fig. 5a, b]. On the other hand, the inhibition of Tfh cells by M-MDSC was insignificant. The percentage of Tfh cells changed slightly under co-culture with M-MDSC in the presence and in the absence of anti-PD-L1 [*F*_(3, 8)_ = 3.072, *P* = 0.091; Fig. 5c, d].

## Discussion

Many types of immune cells are involved in the host immune response to *S. japonicum* infection, such as Th1, Th2, Th17 [15–17], Tfh [18, 19], Treg cells [20, 21], DCs [22–24] and γδ T cells [25]. Tfh cells are a fairly recently identified subset of CD4^+^ T cells that have a distinct gene expression profile and act independently of Th1, Th2 and Th17 cell lineages [26]. There is some supporting evidence that the number of Tfh cells increases in some cases of infectious disease such as immune-active chronic hepatitis B and hepatitis C [27, 28]. According to a very recent report, Tfh cells increased and promoted the formation of hepatic granulomas in mice infected with *S. japonicum* [6]. However, the role of Tfh cells in schistosome infections has not been fully defined. Study of the specific phenotypes and basic immunologic characteristics of Tfh cells is necessary to further elucidate and understand the development and functions of Tfh in various infectious diseases. In this study, we researched the dynamics of Tfh cell formation induced by different antigens of *S. japonicum* in infected mice. Moreover, we found that MDSC can suppress Tfh proliferation. Our results suggest that there are similar trends between the expression of PD-1 on Tfh and PD-L1 on MDSC. We hypothesized that MDSC might regulate the proliferation of Tfh cells through the PD-1/PD-L1 pathway.

In the present study, the proportion of Tfh cells in the spleen increased slowly for 3 weeks post-infection, then increased more rapidly from 3 to 8 weeks, reaching a peak at 8 weeks, and then decreased gradually. Meanwhile, the proportion of Th1 cells among CD4^+^ T cells increased gradually with duration of *S. japonicum* infection, reaching a peak at 5 weeks and then decreasing gradually. The proportion of Th2 cells among CD4^+^ T cells increased gradually with duration of *S. japonicum* infection. Two important antigens (SEA and SWA) are exposed to the host during *S. japonicum* infection [29]. They are both able to induce Th1, Th2 and Tfh cells. Therefore, we immunized mice with SEA or SWA and stimulated CD4^+^ T cells in vitro to confirm the above hypothesis, i.e. that MDSC might regulate the proliferation of Tfh cells through the PD-1/PD-L1 pathway. Our results showed that both SEA and SWA can induce the generation of Tfh cells.

MDSC are a group of heterogeneous cells derived from bone marrow and are precursors of DCs, macrophages and/or granulocytes. MDSC have strong immunosuppressive and immunomodulatory effects and play an important role in tumour immune escape. Recent evidence suggests that MDSC also regulate immune responses in bacterial and parasitic infections, acute and chronic inflammation and autoimmune diseases [30–34]. Studies have shown that MDSC can interact with T cells directly or indirectly. It has been reported that MDSC can promote the differentiation of Th cell subsets by inducing arginase 1 (ARG1) and ARG2 production [35], or by upregulating nitric oxide synthase 2 [36], PD-L1 [37], IL-10 and transforming growth factor beta. It has been confirmed that cytokines secreted by MDSC can promote the differentiation of Treg [38–42] in hematopoietic stem cell transplantation, chronic hepatitis C infection, inflammatory bowel disease and other diseases. However, to our knowledge, whether MDSC regulates Tfh cells in schistosomiasis has not yet been reported. Our study showed that MDSC could suppress Tfh cell proliferation in vitro. Previous research suggested that MDSC could suppress immunity by PD-L1 in human cancers [43, 44]. The ligand PD-L1 interacts with receptor PD-1, which transmits an inhibitory signal mediating the function of these cells [45]. Zeng et al. [46] revealed that a PD-L1 blockade was associated with enhanced accumulation of Tfh cells, and upregulated expression of inducible T-cell co-stimulator and PD-1.

Our data showed that the expression of PD-1 on Tfh cells increased continuously for 8 weeks post-infection, as did the expression of PD-L1 on MDSC cells. There was a similar trend between the expression of PD-1 on Tfh and the expression of PD-L1 on MDSC in mice infected with *S. japonicum*. When G-MDSC or M-MDSC were co-cultured with naïve CD4^+^ T cells in the Tfh condition, the inhibition of G-MDSC on Tfh could be blocked by anti-PD-L1 blocking antibodies. M-MDSC did not significantly inhibit Tfh generation.

The combination of PD-L1 and PD1 can prevent the activation, proliferation and immune response of T cells. The data presented here strongly support the hypothesis that G-MDSC may regulate Tfh cells by PD-L1/PD-1 modulation, which lays a foundation for the important role of Tfh in the immune response to infectious diseases. Additional studies are required to provide more experimental evidence to verify and clarify the comprehensive mechanisms involved in regulation by G-MDSC. In vivo experiments are currently being planned to confirm that MDSC can regulate Tfh cells by PD-L1/PD-1 modulation.

## Conclusions

Our study provides information on the kinetics of the generation of Tfh cells in *S. japonicum*-infected mice. In addition, our data show that, in *S. japonicum* infections, G-MDSC may suppress Tfh cell proliferation through the PD-1/PD-L1 pathway.

## Data Availability

The datasets supporting the conclusions of this article are included within the article.

## Abbreviations

MDSC: Myeloid-derived suppressor cells
PD-1: Programmed cell death protein 1
PD-L1: Programmed cell death ligand 1
SEA: Soluble egg antigen
SWA: Soluble worm antigen
Tfh: T follicular helper
